# The differences of Slovenian and Italian daily practices experienced in the first wave of covid-19 pandemic

**DOI:** 10.1186/s12889-022-12664-5

**Published:** 2022-02-16

**Authors:** Saša Pišot, Boštjan Šimunič, Ambra Gentile, Antonino Bianco, Gianluca Lo Coco, Rado Pišot, Patrik Drid, Ivana Milovanović

**Affiliations:** 1 Institute for Kinesiology Research, Science and Research Centre Koper, Koper, Slovenia; 2 Department of Psychology, Educational Science and Human Movement, Università Degli Studi Di Palermo, Palermo, Italy; 3 Faculty of Sport and Physical Education, University of Novi Sad, Novi Sad, Serbia

**Keywords:** Physical activity and inactivity behaviour, Dietary/eating habits, Well-being, Home confinement, COVID-19 pandemic measures

## Abstract

**Background:**

The COVID-19 pandemic situation with the lockdown of public life caused serious changes in people's everyday practices. The study evaluates the differences between Slovenia and Italy in health-related everyday practices induced by the restrictive measures during first wave of the COVID-19 pandemic.

**Methods:**

The cross-sectional cohort study examined changes through an online survey conducted in nine European countries from April 15–28, 2020. The survey included questions from a simple activity inventory questionnaire (SIMPAQ), the European Health Interview Survey, and some other questions. To compare difference in changes between European countries we examined Italy with severe and its neighbour country Slovenia with low incidence and victims of COVID-19 epidemic. 956 valid responses from Italy (*N* = 511; 50% males) and Slovenia (*N* = 445; 26% males) were investigated.

**Results:**

During the survey, there was a 4.7-fold higher incidence and 12.1-fold more deaths (per 100,000) in Italy than in Slovenia. Barring periods and measures were similar, the latter more stringent in Italy. We found more changes in Italy than in Slovenia: physical inactivity increased (Italy: + 65% vs. Slovenia: + 21%; *p* < 0.001), walking time decreased (Italy: -68% vs. Slovenia: -4.4%; *p* < 0.001); physical work increased by 38% in Slovenia (*p* < 0.001), and recreation time decreased by 37% in Italy (*p* < 0.001). Italians reported a decrease in quality of general health, fitness level, psychological well-being, quality of life and care for own health (*p* < 0.001); Slovenians showed a decline in psychological well-being and quality of life (*p* < 0.001) but generally had a higher concern for their own health (*p* = 0.005). In pooled participants, changes in eating habits (meal size and consumption of unhealthy food), age and physical inactivity were positively correlated with increases in body mass, while changes in general well-being and concern for health were negatively correlated.

**Conclusion:**

The study shows that the negative impact of COVID -19 measures is greater in Italy where the pandemic COVID -19 was more prevalent than in Slovenia with low prevalence. Additional consideration should be given to the negative impact of COVID-19 measures on some health-related lifestyle variables when implementing further measures to mitigate the COVID-19 pandemic.

## Introduction

Temporal rhythms are actively reproduced in everyday life and are understood as accumulations of everyday practices, as performances of coordinating and stabilizing relations between practices [1]. In everyday life, we need to prioritize where routines, habits, and practices are autonomous in order to manage and bind our lives. The rhythms of life give us a sense of security and keep us in constant relationships—this is most evident when our routines and habits are disrupted [2]. The state of COronaVIrus Infectious Disease 2019 (COVID-19) pandemic measures as such a disruptive circumstance, affecting daily practices and disrupting "elementary" routines in such a way that it had a profound effect on overall social integration [3, 4].

In the cross-sectional cohort study in nine European countries, we faced two different situations. Slovenia (SLO) borders Italy (ITA), where citizens witnessed a very serious situation in the first wave of the pandemic in the province of Bergamo, while SLO, in contrast, experienced a "milder" situation as other eastern countries (Slovakia, Croatia and Serbia). This intrigued us to find out how the more serious situation and the higher number of victims, and the resulting severity of the measures taken by Italy compared to Slovenia, affected some lifestyle variables.

The first confirmed case of COVID-19 in ITA was on January 31, 2020, and when further cases emerged in Codogno (Milan), the ITA government imposed a quarantine on infected individuals, their contacts, and those returning from China, on February 21, 2020. Subsequently stricter measures were imposed for northern ITA on March 8, 2020 “Declaration of Red Zones” and a total lockdown on public life “#stayathome regulation” on March 11, 2020. From March 20, 2020 was forbidden to enter in public parks, playground areas, and to play outside. Sports activities were only allowed near residences [5].

The Slovenian COVID-19 experience has been different, emerging about 20 days later with lower incidence and fewer victims as well as milder movement restrictions. After the first case of COVID-19 was confirmed on March 4, 2020 and due to the very difficult situation in Bergamo province (ITA), the SLO government passed the Decree on the Declaration of Contagious Disease SARS-Cov-2 (COVID-19) on March 12, 2020 declaring the first measures: shut down of kindergartens, schools, universities, and from March 16 of open playgrounds, sport parks, sport training camps, and public transportation. As of March 20, 2020 it was forbidden to enter public areas and from March 30, 2020 mobility was only allowed within the municipal borders of residential cities [6].

**Fig. 1:**
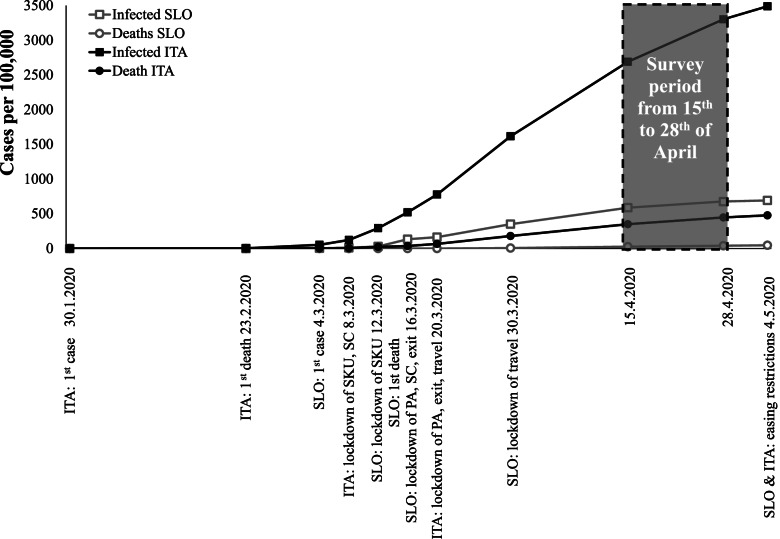
A timeline of restrictions with COVID-19 cases and death in the year 2020 for Italy and Slovenia

SLO measures were initiated later, otherwise comparable to ITA, except for the restriction living the residential and to be physically active, which were stricter in ITA [7]. However, by April 28, 2020, there were 142 times more COVID-19 cases (per capita: 4.9 times) and 325 times more COVID related deaths (per capita: 11.2 times) in ITA than in SLO, and this fact had a significant impact on stricter adherence to restrictions on ITA (Fig. 1) and changed people's everyday life activities more.

It is well known that restrictive measures due to COVID-19 impacted people’s everyday habits [3, 4], however, it is not known how these restrictions impacted two neighbouring countries (SLO and ITA), where COVID-19 restrictive measures lasted for two months with a much worse epidemiological outcome in ITA (especially Northern) than in SLO. Specifically, the quarantine was considered one of the most helpful measures to contain the infection, but on the other hand limited people’s movement outdoors and reduced physical activity mostly to the domestic area.

Because the study examined everyday practices, the research focused on practices that are important to maintain and enhance health, such as domains of physical activity (PA) and, dietary/eating habits that reflect an individual's overall well-being [8]. As smoking and alcohol consumption have been found to increase during the COVID-19 pandemic in some EU countries (Poland and Belgium) [9, 10], these two health-related risk behaviours have also been examined. The positive effects of PA habits on quality of life (QoL) have been demonstrated many times. Not only in active older patients in physical and psychological QoL [11] but also in younger age groups [12], and in chronic patients [13]. PA improves health related QoL in cancer patients [14] and patients with diabetes mellitus [15], where those who met weekly moderate and vigorous PA recommendations reported better physical functioning and were more likely to maintain their physical and overall QoL over time [8]. Covid-19 pandemic studies also suggest an impact on dietary behaviour [4, 16]. Feeling forced to stay indoors because of the lockdown of public life or quarantine could be considered a psychological risk factor for consuming a higher quantitative intake and/or poorer quality (unhealthier) food compared to normal living conditions [17]. The pandemic period could therefore cause changes in dietary habits and energy balance proportions, usually leading to weight gain [17], which is also reflected in QoL.

The aim of the study was to examine the consequences on selected health-related lifestyle variables during COVID-19 restrictions in two neighbouring countries with very different COVID-19 epidemiological impact during COVID-19 first pandemic wave. In light of this, we hypothesized that changes of everyday life practices, such as PA habits, diet (weight gain), health related risk behaviours (alcohol consumption and smoking) and quality of life (QoL) domains were higher in ITA than in SLO due to restrictive government interventions and the severity of COVID-19 epidemiological impact in ITA.

## Methods

### Participants

Altogether 956 Slovenian and Italian respondents were taken for this analysis. See Table 1 for detailed description of the study sample. All respondents were older than 18 years. Informed consent was obtained from all participants on the first page of survey. Specifically, after reading the description of the survey and when they progressed to the first survey question, they marked their consent to participate in the study. See detailed description in Ethics approval and consent to participate. The study was ethically approved by the Faculty of Sport and Physical Education at University of Novi Sad, Serbia (Decision No. 46–06-02/20).

**Table 1:** Sample characteristics

	**Slovenia**		**Italy**	
	***N***	**%**	***N***	**%**
**Sex**				
Males	117	26.3	258	50.5
Females	328	73.7	253	49.5
**Age**				
≤ 20 years	23	5.2	58	11.3
21–30 years	96	21.6	301	58.9
31–40 years	89	20.0	75	14.7
41–50 years	95	21.3	35	6.8
51–60 years	85	19.1	31	6.1
61–70 years	47	10.6	11	2.2
71–80 years	9	2.0	0	0
≥ 81 years	1	0.2	0	0
**Level of education**				
Basic (elementary) education or less	5	1.1	10	2.0
Vocational training	23	5.2	4	0.8
Secondary education	133	29.9	202	39.5
Higher professional education	47	10.5	11	2.2
Higher professional or university education	148	33.3	142	27.8
Master's Degree (Specialist in HE)	46	10.3	127	24.8
Doctoral degree (PhD)	43	9.7	15	2.9

### Research design

This study is a part of a larger cross-sectional cohort study of everyday life practices in the time of the COVID-19 pandemic (ELP-COVID-19 survey) [3, 18] conducted in nine European countries, besides ITA and SLO, also Bosnia and Herzegovina, Croatia, Greece, Kosovo, Serbia, Slovakia, and Spain, from April 15 to 28 2020, with the aim to identify the changes of everyday life practices and routines during the period before (baseline) and during the COVID-19 pandemic measures. ELP (Everyday life praxis) COVID-19 (ELP COVID-19) consortium of six partners from Science and Research Centre Koper (Slovenia), Faculties of sport, University of Novi Sad (Serbia), University of Palermo (Italy), University of Zagreb (Croatia), University of Prešov (Slovakia), University of Cadiz (Spain) has been established for this purpose [3].

### The online questionnaire

The questionnaire “Everyday life in the time of COVID-19 pandemic restriction” (ELP COVID-19 study) was made for the purpose of the research and consisted of whole or/and the adapted parts of validated questionnaires: SIMPAQ – Simple Physical Activity Questionnaire [19] to collect data on sleeping time, PA, inactivity time as time before COVID–19 pandemic (BDC) and time during COVID– 19 pandemic measures (during); adapted part of EHIS European Health Interview Survey [20] for scales to assess eating habits and indicators of quality of life. We assessed the change in body mass (in kg) in those using a body mass scale regularly. For those who did not, we assessed the change of body mass using a 5-point Likert scale (1- deceased a lot, 2- decreased a little, 3- stayed the same, 4- increased a little, 5- increased a lot). Additionally, changes in quality and quantity of eating and other health-related risk habits (alcohol and tobacco use) were also assessed by 5-point (Likert) scale (1-much less, 2-less 3- the same, 4- a little more, 5- much more, another option 6- can't estimate and 7- not applicable (for those who do not consume alcohol and smoke).

The open ended on-line survey consists of a total of 26 questions and was translated from English into eight different languages of participating countries, including Slovene and Italian.

The survey was based on a rendom sample, in which consortium researchers invited participants aged 18 and older who could be reached through a variety of means: personal email addresses, official websites of partner organizations, local online newspapers, etc. Prior the fielding the survey, the electronic questionnaire was tested in all participating countries by the research team in all languages. This included also checking the linguistic and formal suitability of the questionnaire and making any necessary adjustments. The survey was open from 15 to 28 April 2020.

The survey was formed in 1KA, an open-source application that enables services for online surveys, developed by the Centre for Social Informatics, at the Faculty of Social Sciences, University of Ljubljana, Slovenia (https://www.1ka.si/d/en/about/general-description). Data collection and analyses followed the General Data Protection Regulation (GDPR). Participation in the survey was voluntary, visitors of the first page can decide to participate after the survey announcement. Additionally, respondents had the option to opt out of the questionnaire at any point prior to the submission process. Respondents were able to review and change their answers using the back button. Most of the questions in the survey were mandatory, a check for completeness after submitting the questionnaire was possible and mandatory items were highlighted.

### Statistics

Only surveys with completed mandatory questions were taken into analysis. The SPSS (version 26.0, IBM, USA) was used for data analysis. All data were presented as mean (standard deviation) values and were analysed separately for SLO and ITA regarding the collected data for the times before and during COVID-19 pandemic measures. Normal distribution (Histogram, Q-Q-plot, Skewness, Kurtosis, Shapiro–Wilk test) and homogeneity of variance (Levene test) were checked and met. The multivariate difference in all 14 everyday practices variables were tested by multivariate Hotelling's T^2^ test [21], while differences in each variable was tested by 2-way ANOVA (time, country) with age and sex differences between countries as a covariates. Statistical significance was set at *p* < 0.05. Where changes in baseline values (before) were identified, an analysis of covariance (ANCOVA), with baseline values as additional covariate, was used to determine differences in changes during COVID-19 restrictions. Identification of significant predictors of body mass changes in the subsample of those using a body mass scale regularly (*N* = 548) was made by a Multiple Linear Regression, where predictors passed non-multicollinearity assumptions (variance inflation factor < 2). A subsample was used to assure interval scale of dependent variable (body mass change). Additionally, we presented frequency analysis of gaining weight (for a subsample of those who did not monitoring weight, *N* = 408), changes in eating habits, alcohol consumption and smoking in Fig. 3. For that purpose, only subjects that responded on a 5-point (Likert) scale: 1-much less, 2-less, 3-the same, 4-little more, and 5-much more, were analysed: While those that indicated 6-cannot estimate or 7-not applicable were excluded. In each analysis (of practices) a Bonferroni correction of p-value was used.

## Results

### Description of the study sample

Respondents answered the survey over a period of 33.9 (6.0) days after pandemic measures were declared by the state government in SLO and 44.9 (6.4) days in ITA, when most changes could become latent. The participation rate was 17% in SLO and 19% in ITA while the completion rate reached 31% in SLO and 73% in ITA. The sample (Table 1) consisted of 445 SLO (26.3% males, aged 42.1 ± 14.9 years) and 511 ITA respondents (50.5% males, aged 29.6 ± 11.0 years). Most of the respondents were between 21–30 years old in ITA and 21–60 years in SLO, respectively, and representing the active population (employed, self-employed) (SLO 67%, ITA 46.5%) and students (SLO 16.4%, ITA 40.5%). Most respondents had a higher level of education and especially in the ITA sample prevail younger population, as the primary dissemination channel of the online survey was among students and university staff.

**Fig. 2:**
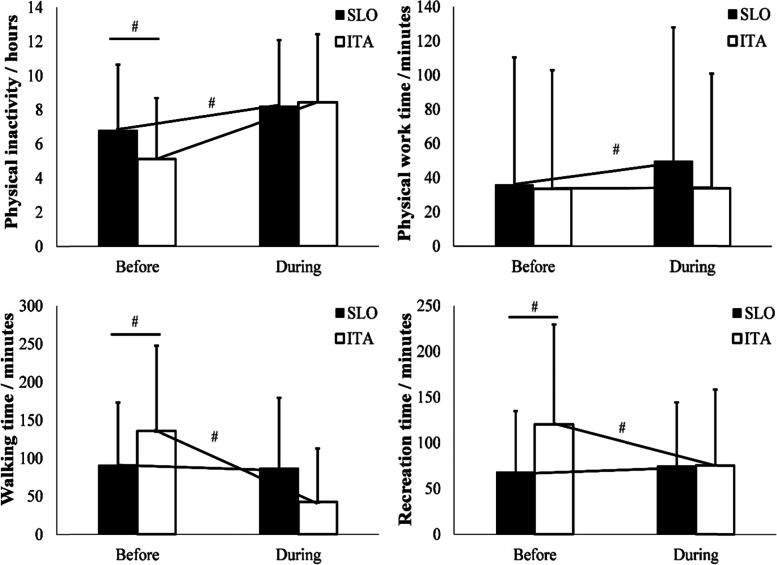
Physical inactivity, Physical work, Walking time and Physical activity as recreation time before and during COVID-19 measures

Baseline physical inactivity differed between the countries; therefore, we controlled different baseline values using ANCOVA, and confirmed greater changes in ITA than in SLO (Fig. 2). Specifically, physical inactivity increased (ITA 65% vs. SLO 21%; *p* < 0.001); physical work increased only in SLO by 38% (*p* < 0.001); walking time decreased (ITA 68% vs. SLO 4.4%; *p* < 0.001); however, sport recreation time decreased in ITA by 37% (*p* < 0.001) but increased in SLO by 9.7% (*p* < 0.001).

**Fig. 3:**
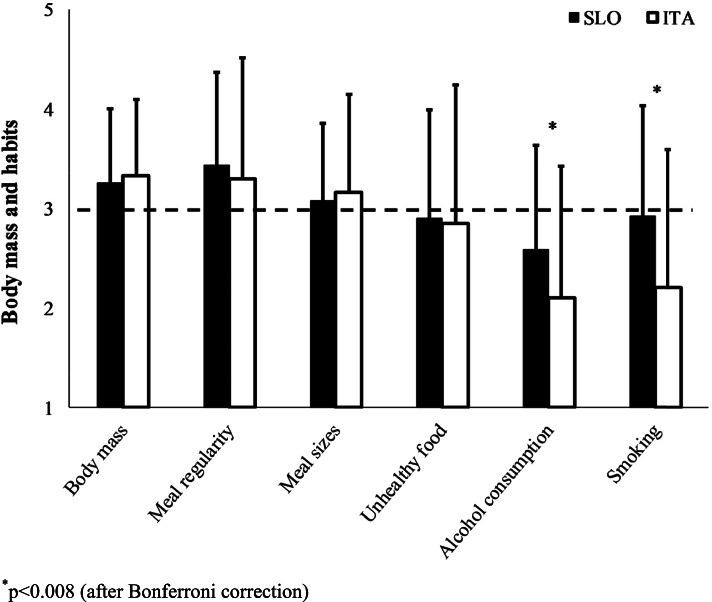
Likert scale values of body mass, eating habits, alcohol consumption, and smoking changes (increases > 3 or decreases < 3) during COVID-19 restrictions in Slovenia (SLO) and Italy (ITA)

Figure 3 presents changes in nutrition during COVID-19 restrictions in ITA resulting in an increase of body mass, meal regularity and meal sizes, while alcohol consumption and smoking decreased (*p* < 0.001). Similarly, in SLO COVID-19 restrictions increased body mass and meal regularity while alcohol consumption decreased (*p* < 0.001). The difference in the magnitude of change between the two countries was found in alcohol consumption decreasing more in ITA than in SLO (2.11 ± 1.32 vs. 2.59 ± 1.04; *p* = 0.001) and in smoking, which decreased only in ITA, while in SLO it remained unchanged (2.21 ± 1.39 vs. 2.93 ± 1.11; *p* < 0.001).

**Fig. 4:**
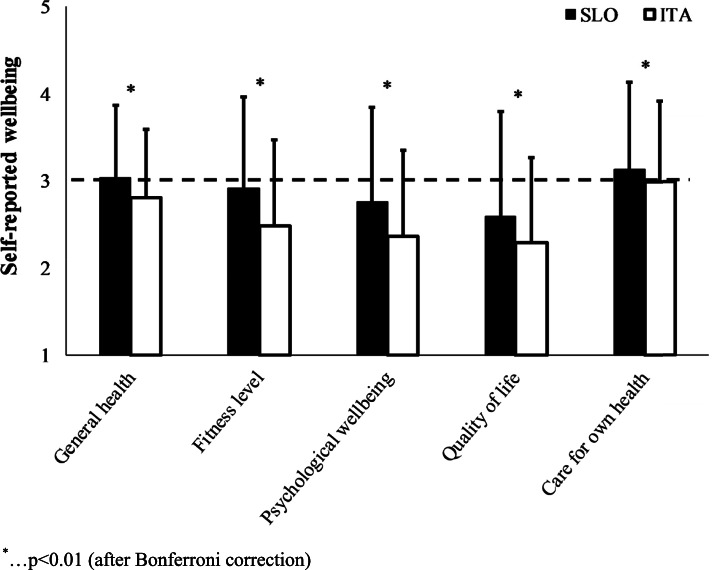
Likert scale values of self-reported wellbeing changes (increases/better > 3 or decreases/worse < 3) during COVID-19 restrictions in Slovenia (SLO) and Italy (ITA)

Figure 4 presents changes in self-reported wellbeing during COVID-19 restrictions. ITA reported worse general health status, fitness levels, psychological wellbeing, quality of life and care for own health (*p* < 0.001). COVID-19 restrictions in SLO worsened psychological wellbeing and quality of life (*p* < 0.001), but increased care for own health (*p* = 0.005). Differences in the magnitude of change between the countries were found in general health (*p* < 0.001) and fitness levels (*p* < 0.001), which decreased only in ITA, while psychological wellbeing (*p* < 0.001) and quality of life (*p* < 0.001) decreased more in ITA than in SLO.

Additionally, we analysed a subsample of 548 participants who used body mass scale regularly and quantitatively reported changes of body mass in the range of -7 to + 7 kg with an average increase of 0.27 ± 2.02 kg where a multiple linear regression explained 22% (*R* = 0.470; *p* < 0.001) of body mass change variance through seven predictors (Table 2). Increase of meal sizes, unhealthy food consumption, psychological wellbeing, age, and physical inactivity positively contributed to body mass gains, while general health and care for own health contributed negatively to body mass gains.

**Table 2:** A multiple linear regression of body mass increase during COVID-19

Predictor	B	β	*p*	Partial R	VIF
Constant	-5.470		0.283		
Meal sizes	0.492	0.226	< 0.001	0.234	1.15
Unhealthy food	0.251	0.160	< 0.001	0.167	1.15
General health	-0.399	-0.165	0.001	-0.141	1.72
Psychological wellbeing	0.224	0.116	0.014	0.108	1.45
Care for own health	-0.381	-0.183	< 0.001	-0.167	1.51
Age	0.010	0.076	0.047	0.084	1.03
Changes in physical inactivity	0.001	0.065	0.058	0.071	1.08

Dependent variable: body mass change from before to during COVID−19 pandemics; B…regression coefficient; β…standardised regression coefficient; Partial R…partial correlation coefficient, *VIF* Variance inflation factor

## Discussion

The current paper represents an upgrade of the cross-sectional cohort  study ELP-COVID-19 survey [3] conducted among 4108 participants from nine European countries. It compares two countries, SLO and ITA, with different restrictive measures and very different epidemiological outcomes of the COVID-19 pandemic [3].

We confirmed that the restrictive COVID-19 pandemic measures in the 1^st^ wave that lasted about two months (SLO: from March 12 to May 4, 2020; ITA: from March 8 to May 4, 2020) with associated higher daily new cases and deaths in ITA had a grater impact on ITA than SLO. From the World Health Organization Coronavirus Disease (COVID-19) dashboard [7] and Fig. 1 it is evident that in the period of the active online survey, from April 15 to 28 2020 ITA had more COVID-19 cases, rose from 162,488 to 199,414 cases, than in SLO, rose from 1,220 to 1,407 cases. A similar difference was observed in COVID-19 related deaths, where in ITA they rose from 21,069 to 26,977 (an increase of 5,908 deaths) and in SLO from 56 to 83 (an increase of 27). On April 28 2020, COVID-19 prevalence among the population was 3.3% in ITA and 0.06% among SLO residents [7]. At the same time we found that this affected everyday life practices in the ITA population more than in the SLO population. COVID-19 related changes were greatest in PA domains (physical inactivity, walking, recreational sports, and physical work).

Increases in physical inactivity and decreases in walking time, recreation time and physical work in ITA were highly variable (37–80%) and greater compared with SLO (from no change to 58%). Due to differences in age and sex distribution between the samples of ITA and SLO (younger population and more females in ITA compared to SLO), we obtained BDC differences between both countries, showing lower physical inactivity in ITA and higher walking time and recreation time in ITA than in SLO. However, these differences were statistically included in the analysis as a covariance to confirm a higher impact in ITA than in SLO. Nonetheless, these BDC differences yielded even more relevant effects of COVID-19 restrictions in ITA than in SLO. Moreover, self-reported general health and fitness levels declined only in ITA, whereas psychological well-being and quality of life declined in both countries. Concern about own health increased only in SLO.

At the first glance COVID-19 consequences in SLO were mitigated most likely due to lower absolute or per capita numbers of new COVID-19 cases and deaths. This is supported by a comparison of restrictive measures, which were similar in both countries. The difference is that in ITA the first major restriction was initiated 38 days after the first COVID-19 case while in SLO only 8 days after the first COVID-19 case. However, SLO also experienced milder COVID-19 pandemic restrictions limiting outdoor movement [6].

When comparing ITA and SLO consequences in the PA domain with pooled data from nine European countries [3], we found a higher increase in physical inactivity in ITA (65%) than in the pooled countries (50%) and lower in SLO (21%). Walking time decreased more in ITA (69%) than in the pooled countries (21%) and in SLO (4.4%). Recreational time also decreased more in ITA (37%) compared to the pooled countries (24%) while in SLO this increased (9.7%). Interestingly, while physical work remained unchanged in ITA, it increased in the pooled countries (37%), similarly to the case in SLO (38%). This can be explained by the differences in the ITA sample, as the younger population is not as involved in physical work activities (e.g., gardening) compared to the Slovenian sample, where the older population was more involved in domestic or external physical work typical of spring [3]. A study [4] using the International Physical Activity Questionnaire Short Form (IPAQ-short) [22] in 22 countries reported similar changes in increasing sitting time (28.6%), decrease of walking (34%) and moderate and vigorous PA decreased by ~ 33%. It is evident also in our study that changes in PA in ITA were significantly greater than in SLO and greater than in the aforementioned study of 22 countries, as reported in the literature [3, 4].

There was an impact of COVID-19 restrictions on eating habits, resulting in an equal increase of body mass in both countries. Twenty-two percent of the variance in body mass gain was explained by larger  meal sizes (seen only in ITA), participant’s age, increased physical inactivity (seen in both countries), lower  self-perception of general health status (seen only in ITA), increased self-perception of psychological wellbeing (seen in both countries), lower  self-perception of care for health (increased only in SLO) and increased unhealthy food intake, although on average this did not change in SLO and ITA. We could speculate that physical inactivity, increased meal sizes, age, and psychological wellbeing could have a causal relationship with body mass gains and general health status with own health care a consequential relationship with body mass gains. Diminished work duties, home confinement, self-scheduling, increased screen time [1, 23] along with stockpiling of food led to increased psychological wellbeing, which in turn may have induced overeating and consequent unbalanced energy intake [24, 25]. Whereas among lifestyle characteristics, regular exercise seems to be the most important independent predictor for a perceived overall health-related quality of life [26, 27], on the other hand a low self-image after drastically increased physical inactivity and body mass gain could explain decreased self-perception of general health status and care for own health [28].

Nevertheless, a “positive” COVID-19 consequence was in decreased alcohol consumption as was seen in other studies [7], however, larger changes were found in ITA than in SLO; smoking, on the other hand, decreased only in ITA. This could be explained by the measures of home confinement and restricted access to public spaces, which reduces social life and the accompanying social habits such as drinking and smoking, that are normally associated with the population of young people who was more represented in ITA sample.

## Conclusions

Our study presents the consequences in some health-related daily practices (PA and dietary habits) of different restriction measures in two neighbouring countries with very different numbers of COVID-19 cases and deaths. Although the restrictions were not drastically different, they had a great effect on what differences occurred between two countries. The smaller differences in everyday life practices were more beneficial to SLO, as ITA population experienced 44% greater increases in physical inactivity, a 64% greater decrease in walking time, a 47% greater decrease in recreation time, and did the same amount of physical work as before Covid-19 restrictions, whereas in SLO it increased by 38%. As the present study highlights the consequences of the two different COVID-19 pandemic scenarios on people’s everyday lives, especially of those that are important for ensuring health, both health related habits, PA and eating habits, and also health risk behaviour (alcohol consumption and smoking) were included in the protocol. The consequences found in our study were previously linked to chronic diseases such as obesity, diabetes, cardiovascular diseases, cancer and others [7, 24], which were also linked to higher mortality rates in COVID-19 patients [29]. Therefore, when setting limits between outbreaks or waves of COVID-19, governments should be aware of the harmfulness of limiting health determinants as a direct consequence of COVID-19 restrictions. Measures and restrictions should be based on scientific grounds and professional guidelines, otherwise even more serious consequences and outcomes can be expected in the event of a recurrence of the disease or the onset of a different one.

## Data Availability

The datasets used and/or analysed during the current study are available from the corresponding author on reasonable request.

## Abbreviations

BDC: before COVID–19 pandemic
“ELP COVID-19” consortium: Consortium of six partners from Science and Research Centre Koper (Slovenia), Faculties of sport, University of Novi Sad (Serbia), University of Palermo (Italy), University of Zagreb (Croatia), University of Prešov (Slovakia), University of Cadiz (Spain)
GDP: General Data Protection Regulation
ITA: Italy
SLO: Slovenia
WHO: the World Health Organisation
